# The business management training needs of South African Biokineticists to ensure business sustainability

**DOI:** 10.17159/2078-516X/2021/v33i1a9220

**Published:** 2021-07-05

**Authors:** MJ Lumb, K Marais

**Affiliations:** School of Management, IIE’s Varsity College, Belmont Office Park, Belmont Rd, Rondebosch, Cape Town, 7700, Western Cape, South Africa

**Keywords:** administration, education, health professionals, knowledge, practice management, skills

## Abstract

**Background:**

Business management training is essential for success in the modern era. Health and medical professionals are exposed to knowledge that allows them to treat pathologies. However, their training does not prepare them to manage their practices as businesses and in a sustainable, effective, and efficient manner.

**Objectives:**

To investigate the business management training needs of registered South African Biokineticists.

**Methods:**

A quantitative and descriptive research design was used. Sixty-nine registered Biokineticists answered the emailed survey. The survey was sent out on two separate days, two weeks apart. Participants could only answer the survey once. The survey was sent out by the Biokinetics Association of South Africa (BASA). The sample in this study consisted of both male and female participants who graduated between the years of 1985–2019. The survey consisted of demographic questions about their study methods. It also included a 5-point Likert Scale where a score of 1 indicated an exceptionally low need and a score of 5 indicated a very high need for corresponding business processes. The business processes included accounting, business sustainability, corporate social responsibility, ethics, financial management, human resource management, leadership and managerial decision-making, marketing, operational management, and strategic management. A final open-ended question on what other business management training the participant needed was asked at the end of the survey.

**Results:**

Combined high to very high needs (X≥4 on the Likert Scale) for the business management processes explored were: accounting: 28%, business sustainability: 33%, corporate social responsibility: 23%, ethics: 55%, financial management: 35%, human resource management: 29%, leadership and managerial decision-making: 43%, marketing: 41%, operational management: 39%, and strategic management: 33%. Seventy-one percent of the participants who took part in the study suggested that they needed other business management training needs, providing suggestions in the final question. Of the 71% of participants who answered this question, the most important requests identified included information technology (17%), tax-related management and knowledge (19%) and medical aid training for ICD-10 coding (13%). The other 51% of the participants that answered the final question provided suggestions that could be categorised into the areas of business already reported on in the Likert Scale. Sixty-nine out of a possible ±1600 registered Biokineticists who were BASA members completed the survey. This represents a response rate of about 4%.

**Conclusion:**

Business management training needs exist for South African Biokineticists. By addressing these needs, it may lead to improvements in overall patient care, practice management and small business growth which in return can lead to the socioeconomic stimulation of the country.

The profession of Biokinetics is a specialised discipline in exercise therapy. The name is derived from two Greek words: *Bio*’ meaning life and ‘*Kinesis*’ meaning movement. Therefore, Biokinetics means ‘*life through movement*’. ^[4]^

The healthcare industry currently finds itself undergoing rapid changes that are leading to an ever more competitive environment. Professionals are continuously challenged with balancing the concepts of business management, knowledge development and business sustainability with patient quality of life while trying to maintain ethical integrity. ^[1,5,6,7,8]^

Business management training needs for health and medical professionals are essential as many practitioners may find themselves in leadership and managerial positions within practices and multidisciplinary medical centres.^[1]^ By upskilling medical and health professionals with business knowledge it may lead to an increase in local entrepreneurship which could benefit a struggling economy such as South Africa. It may also lead to the reduction of professionals seeking work overseas if more local work opportunities are available.^[5,9]^ To our knowledge, few studies around the world have determined the business management training needs of health and medical professionals and no study of this sort has been performed on South African Biokineticists.

Similar studies have been performed on other health and medical professions around the world and in South Africa. ^[1,5,6,7]^ These studies have displayed a need for a more in-depth approach to teaching business concepts to professionals such as Chiropractors and Audiologists. ^[1,5]^ Table 1 tabulates the high to very high needs for South African Biokineticists, South African Audiologists and North American Chiropractors ^[1,5]^. The results of the above three studies are not directly comparable due to the current study using a 5-point Likert-scale (very low, low, moderate, high and very high) compared to the 4-point Likert-scale used in the Audiologist and Chiropractic studies (very low, low, high, very high). However, the ranking of importance for the business management fields can be compared ^[1,5]^.

The question now arises whether there is sufficient business management training for Biokineticists which equips them to run a successful and sustainable business.

## Methods

### Study design

**Fig. 1:**
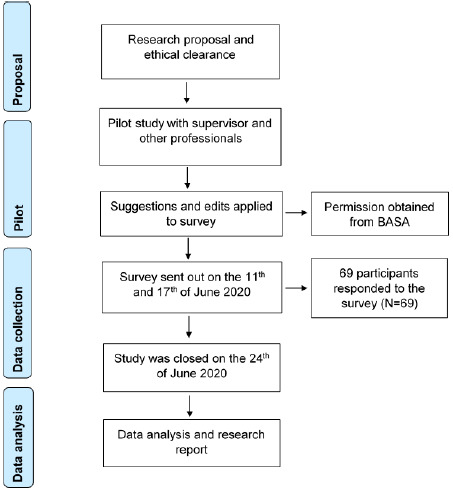
Research design (Adapted PRISMA Flow Diagram) (Moher, Liberati, Tetzlaff, Altman & The PRISMA Group, 2009) ^[10]^

A quantitative and descriptive research design was conducted with the use of an online-based survey. The survey was distributed via email by the Biokinetics Association of South Africa (BASA) to registered Biokineticists. The first email was sent on the 11^th^ of June 2020 and the second email was sent on the 17^th^ of June 2020. Participants were limited to one opportunity to answer the survey. The study was terminated on the 24^th^ of June 2020. The study protocol was approved by the Campus Research Postgraduate Studies Committee at the Cape Town IEE’s Varsity College Campus. Fig. 1 is a schematic illustration of the research design that was followed in the study.

The population used in this study were South African Biokineticists who were members of the Biokinetics Association of South Africa (BASA). A non-randomised convenience sampling method was implemented. Participants had to be fully registered with the HPCSA and members of BASA. Intern Biokineticists were excluded from the study. Prospective participants were emailed an information letter and a link to the online-based survey. Informed consent was built into the online survey. The survey could not be completed unless informed consent was given. Potential participants had to read the information letter and agree to the study by initialising a required question: “Do you consent to partake in the following study? If so, please type in your initials below (Information & consent form above)”. If this question was not completed, the potential participant would not be able to proceed further with the survey.

A sample of 69 individuals was included in descriptive reporting. The participants were taken from a wide graduation period. They were grouped according to the graduation period as detailed in Table 2. ‘Graduation period’ is defined as the time in which a participant graduated with their Biokinetics degree. The year 2020 was not included as students still needed to complete a year of internship to fully qualify.

### Study procedure

Participants were emailed an information letter which provided the details of the study and a link to the online-based survey. The online-based survey was designed by consulting previous literature and studies that explored similar needs amongst North American Chiropractors and South African Audiologists.^[1,5]^

### Data collection methods

The data collection method used in this study was an online-based survey that was sent to BASA registered Biokineticists via email. The survey was sent as a link to possible respondents who had to read the attached information letter and the consent form. The survey was created with Microsoft Forms. ^[11]^ Respondents had to follow the instructions and answer the questions that followed. This type of data collection method was ideal for the current circumstances of practising social distancing due to COVID-19. The method was also cost-effective for the researcher and it allowed the respondents to complete the survey in their own time. The first email was sent out on the 11^th^ June 2020. A reminder email was sent out to on the 17^th^ June 2020. The study was closed on the 24^th^ June, which was two weeks after the initial email was sent. A coding technique was implemented to filter out participants who responded more than once.

### Data collection tool

The survey that was sent to BASA registered Biokineticists included questions about their graduation period. A 5-point Likert Scale was used for responses to questions on business training where 1 was very low, 2 was low, 3 was moderate, 4 was high and 5 was very high. There was an open-ended final question on any other business knowledge training.

Before sending out the survey, a pilot survey was conducted with multiple different professionals. This was done to receive a broad spectrum of feedback in order to eliminate as many errors as possible.

The data collection tool was designed by consulting previous studies that explored similar topics in the medical field.^[1,5,6]^ All survey questions were compulsory and none could be left unanswered.

### Statistical analysis

The level of confidence for the research was set at 95% (p≤0.05). The research made use of descriptive statistics and measures of central tendencies, including means and standard deviations. It also included frequencies and ranking of the results. The data were analysed in Microsoft Excel with the use of its built-in data analysis add-on pack. Ranking is the sum of high and very high percentages. Rank 1 is the highest knowledge need amongst South African Biokineticists. Equal high to very high knowledge needs were given equivalent ranking.

## Results

The distribution for the needs of the following business concepts is detailed in Table 3.

The final open-ended question asked if there were other business management training needs that were not mentioned in the survey or could not be categorised with the already mentioned business processes. The answers were examined by the researcher and categorised into three main categories: technology management (n=8, 17%), tax management (n=9, 19%), and ICD/medical aid training (n=6, 13%).

## Discussion

The current study explored the business knowledge training needs for BASA-registered Biokineticists. Even though the data presented was only descriptive, it provides insight into areas where the profession needs guidance and further education. This study can be used as a pilot for future studies that explore similar topics, as well as for the future development of the Biokinetics curriculum. The importance of a successful medical, health and fitness industry in South Africa cannot be underestimated as a driver for social and economic development.^[12]^ An improvement in and development of the managerial skills of the practitioners may make them better suited to implement successful business strategies in addressing the burden of disease that impacts a developing country like South Africa.^[12]^ Furthermore, the study may assist practitioners to have a more impactful and successful outcome when addressing the needs of the population if the limited scope and quality of management they are taught is addressed.^[12]^

In accordance with previous studies, the survey was designed to determine the current needs of South African Biokineticists across a broad spectrum of business processes.^[1,5,6,7,8]^ The study included two newer schools of thought within the business environment, namely corporate social responsibility and business sustainability which are critical to the success of any modern business.^[13]^ Also, these two concepts were not explored in previous studies.^[1,5,6,7]^ Without a thorough understanding of these concepts, the practitioner may end up neglecting fundamental stakeholders and within their business itself.^[13]^ By exposing health and medical professionals to concepts such as business sustainability and corporate social responsibility, practitioners can better position themselves to assist the country in addressing the initiatives of encouraging physical activity and healthy lifestyles.^[12]^ It can also provide unique opportunities for practitioners to involve themselves in advising and managing government initiatives that are aimed at addressing chronic and lifestyle diseases, the type of facilities that are required, and the direction in which the industry should go.^[12]^ Biokineticists have a unique opportunity to evolve the profession and address the above-mentioned gaps in the market.

Large market demands are present in South Africa for Biokineticists; however, to successfully access these market demands the correct business knowledge, training and skills are required. ^[14,15]^ Biokineticists are left with limited career choices within South Africa, often finding themselves working for, or opening, private practices ^[14]^. The prospect of succeeding with these options are slim in South Africa, where the small business failure rate is between 70–80% ^[15]^.

### Limitations

Numerous limitations are present in the current study, such as the small sample size, non-randomised sampling implementation, the short duration of the study and the use of an online-based survey, which tends to have low response rates. However, this was the most suitable method due to the regulations in place to limit the spread of COVID-19. The current study did not differentiate between practitioners that worked for practices, managed a practice, owned a practice, or were academics involved in research. Furthermore, the study only included BASA members which could have led to the omission of practitioners only registered with the HPCSA.

In future studies, researchers should attempt to conduct randomised sampling and to apply inferential statistics and inferences from the sample to the population. Furthermore, it will be useful if future studies implement a mixed-methods data collection technique to gain more insight into the type of training and knowledge needed by practitioners. The inclusion of Biokineticists not registered with BASA is also recommended.

Future studies should explore associations between variables, including where practitioners graduated from and the business knowledge needs required, as well as the year in which they graduated.

Furthermore, future studies should perform in-depth analyses of the current Biokinetics curriculum to determine what business skills are being taught.

## Conclusion

Identifying and addressing shortfalls in business knowledge training for healthcare professionals, such as Biokineticists, may lead to improvements in overall patient care and practice management. This may result in the growth of small practices, becoming sustainable and successful in a modern business environment.^[14,15]^ Furthermore, by addressing the abovementioned gaps in education and knowledge, Biokineticists may have an opportunity to evolve and implement themselves in addressing the larger socioeconomic and health-related issues.

## Figures and Tables

**Table 1:** Comparison of business knowledge training needs across studies

Business knowledge	Business knowledge training needs for South African Biokineticists	Required practice management knowledge for South African Audiologists ^[5]^	Business training and education needs of North American Chiropractors ^[1]^

Need	High and very high	High and very high	High and very high
Accounting	28 (8)	92 (3)	79 (6)
Business sustainability	33 (6)	N/A	N/A
Corporate social responsibility	23 (9)	N/A	N/A
Ethics	55 (1)	95 (2)	81 (5)
Financial management	35 (5)	92 (4)	83 (3)
Human resource management	29 (7)	89 (7)	89 (1)
Leadership	43 (2)	90 (6)	70 (7)
Marketing	41 (3)	96 (1)	82 (4)
Operational management	39 (4)	90 (6)	62 (8)
Strategic management	33 (6)	92 (5)	86 (2)

Data are displayed as percentage % (ranking)

**Table 2:** Graduation period count (n)

Graduation period (years)	Count of graduation period (n)
Before 1980	0
1985–1989	1
1990–1994	2
1995–1999	2
2000–2004	15
2005–2009	14
2010–2014	14
2015–2019	21

**Total count (N)**	**69**

**Table 3:** Distribution and ranking of business knowledge training needs amongst South African Biokineticists, rated on a 5-point Likert Scale.

	Likert Scale Rating					

Business knowledge need	Very low (1)	Low (2)	Moderate (3)	High (4)	Very high (5)	Ranking
Ethics	7 (5)	12 (8)	26 (18)	30 (21)	25(17)	1
Leadership and managerial decision- making	9 (6)	22 (15)	26 (18)	30 (21)	13 (9)	2
Marketing	6 (4)	17 (12)	36 (25)	29 (20)	12 (8)	3
Operational management	10 (7)	19 (13)	32 (22)	25 (17)	15 (10)	4
Financial management	3 (2)	36 (25)	26 (18)	25 (17)	10 (7)	5
Strategic management	10 (7)	33 (23)	23 (16)	25 (17)	9 (6)	6*
Business sustainability	12 (8)	22 (15)	33 (23)	25 (17)	9 (6)	6*
Human resource management	12 (8)	26 (18)	33 (23)	25 (17)	4 (3)	7
Accounting	19 (13)	19 (13)	35 (24)	23 (16)	4 (3)	8
Corporate social responsibility	15 (10)	23 (16)	39 (27)	17 (12)	6 (4)	9

Data are displayed as percentage % (n). Ranking is the sum of high and very high percentages.

* tied rankings between strategic management and business sustainability.

## Glossary

Accounting: The business process that is concerned and focused on managing, recording, reporting, and analysing financial transactions of an organisation ^[1]^
Business sustainability: The business process that concerns itself with the impact the organisation has on internal and external stakeholders. It is comprised of three main elements including social, environmental, and economic ^[2]^
Corporate social responsibility: The business process that is concerned with integrating business, social and environmental factors from the strategic level to all levels of the organisation so to achieve a sustainable future for present and future stakeholders ^[2]^
Ethics: The business process that concerns itself with ethical decision making and what is considered the correct thing to do at a given point in time. It takes into consideration contracts, laws, and regulations that govern and guide an organisation ^[1]^
Financial management: The business process that is focused on acquiring, investing, and managing all the resources of an organisation including tangible and non-tangible assets ^[1]^
Human resources management: The business process that is focused on the management of people within the organisation and the acquisition of new talent. It is also focused on developing and acquiring knowledge for the organisation, hiring, and firing employees and the development and implementation of the organisation's culture ^[1]^
Leadership and managerial decision-making: Can be described as a complex ever evolving social theory where one or more influential people empower and guide skilled individuals towards achieving a common shared goal ^[3]^
Marketing: The business process that is focused on defining and selecting potential target markets. It is also concerned with the management of the product/service, pricing, and communication ^[1]^
Operational management: The business field focused on managing the processes that produce and distribute a service or product to the organisation's target market ^[1]^
Strategic management: The business process that is focused on identifying and navigating an organisation towards meeting its goals and objectives ^[1]^.
